# Cytokine storms are primarily responsible for the rapid death of ducklings infected with duck hepatitis A virus type 1

**DOI:** 10.1038/s41598-018-24729-w

**Published:** 2018-04-26

**Authors:** Jinyan Xie, Mingshu Wang, Anchun Cheng, Xin-Xin Zhao, Mafeng Liu, Dekang Zhu, Shun Chen, Renyong Jia, Qiao Yang, Ying Wu, Shaqiu Zhang, Yunya Liu, Yanling Yu, Ling Zhang, Kunfeng Sun, Xiaoyue Chen

**Affiliations:** 1 Institute of Preventive Veterinary Medicine, Sichuan Agricultural University, Wenjiang, Chengdu City, Sichuan People’s Republic of China; 2 Key Laboratory of Animal Disease and Human Health of Sichuan Province, Sichuan Agricultural University, Wenjiang, Chengdu City, Sichuan People’s Republic of China; 3 Avian Disease Research Center, College of Veterinary Medicine, Sichuan Agricultural University, Wenjiang, Chengdu City, Sichuan People’s Republic of China

## Abstract

Duck hepatitis A virus type 1 (DHAV-1) is one of the most harmful pathogens in the duck industry. The infection of adult ducks with DHAV-1 was previously shown to result in transient cytokine storms in their kidneys. To understand how DHAV-1 infection impacts the host liver, we conducted animal experiments with the virulent CH DHAV-1 strain and the attenuated CH60 commercial vaccine strain. Visual observation and standard hematoxylin and eosin staining were performed to detect pathological damage in the liver, and viral copy numbers and cytokine expression in the liver were evaluated by quantitative PCR. The CH strain (10^8.4^ copies/mg) had higher viral titers than the CH60 strain (10^4.9^ copies/mg) in the liver and caused ecchymotic hemorrhaging on the liver surface. Additionally, livers from ducklings inoculated with the CH strain were significantly infiltrated by numerous red blood cells, accompanied by severe cytokine storms, but similar signs were not observed in the livers of ducklings inoculated with the CH60 strain. In conclusion, the severe cytokine storm caused by the CH strain apparently induces hemorrhagic lesions in the liver, which might be a key factor in the rapid death of ducklings.

## Introduction

Duck hepatitis A virus type 1 (genus *Avihepatovirus*, family *Picornaviridae*, DHAV-1) is one of the most common and lethal pathogens in young ducklings and is responsible for acute hepatitis, characterized by petechial and ecchymotic hemorrhages of the liver surface^1,2^. The liver, a major site for the regulation of immune and inflammatory responses, plays a critical role in defending against invasive pathogens^3,4^. Immune responses in the liver appear to have evolved to balance virus eradication and immunopathology^5^. Major hepatotropic viruses, such as hepatitis A virus (HAV), hepatitis B virus (HBV), and hepatitis C virus (HCV), interact with innate immunity factors and induce both interferons (IFNs, types I and III) and antiviral IFN-stimulated genes (ISGs), and these viruses have developed multiple strategies to escape innate immune responses^6–10^. Inflammation is a double-edged sword that plays a vital role in liver metabolism. Moderate inflammatory responses confer a certain degree of protection, help repair damaged tissue, and promote steady-state reconstruction. However, uncontrolled inflammatory responses are present in most clinical cases of liver disease and may form a “storm” that causes liver damage, fibrosis, cirrhosis and other adverse consequences^11–13^.

Infection of adult ducks with DHAV-1 was previously shown to result in transient cytokine storms in their kidneys^14^. However, only one study has investigated virus-host interactions in the livers of DHAV-1-infected ducklings. Therefore, in this study, we established an experimental model utilizing the virulent CH DHAV-1 strain and the attenuated CH60 commercial vaccine strain to investigate DHAV-1 pathogenicity and host immune responses in 7-day-old ducklings.

## Results

### Gross lesions

**Figure 1:**
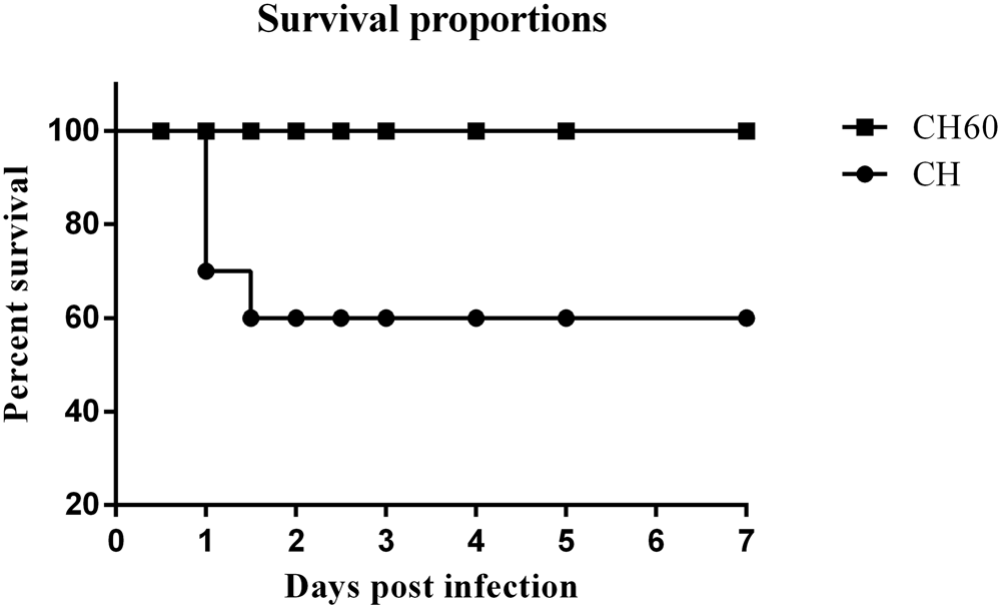
Survival proportions of CH and CH60-inoculated ducklings.

At 24 hours post-infection (hpi) with the CH DHAV-1 strain, the ducklings generally showed typical clinical signs, such as mental depression, anorexia and drowsiness. Mortality occurred within 24–48 hpi (Fig. 1), and the ducklings exhibited typical opisthotonos. However, none of the CH60- inoculated ducklings exhibited clinical signs or died.

**Figure 2:**
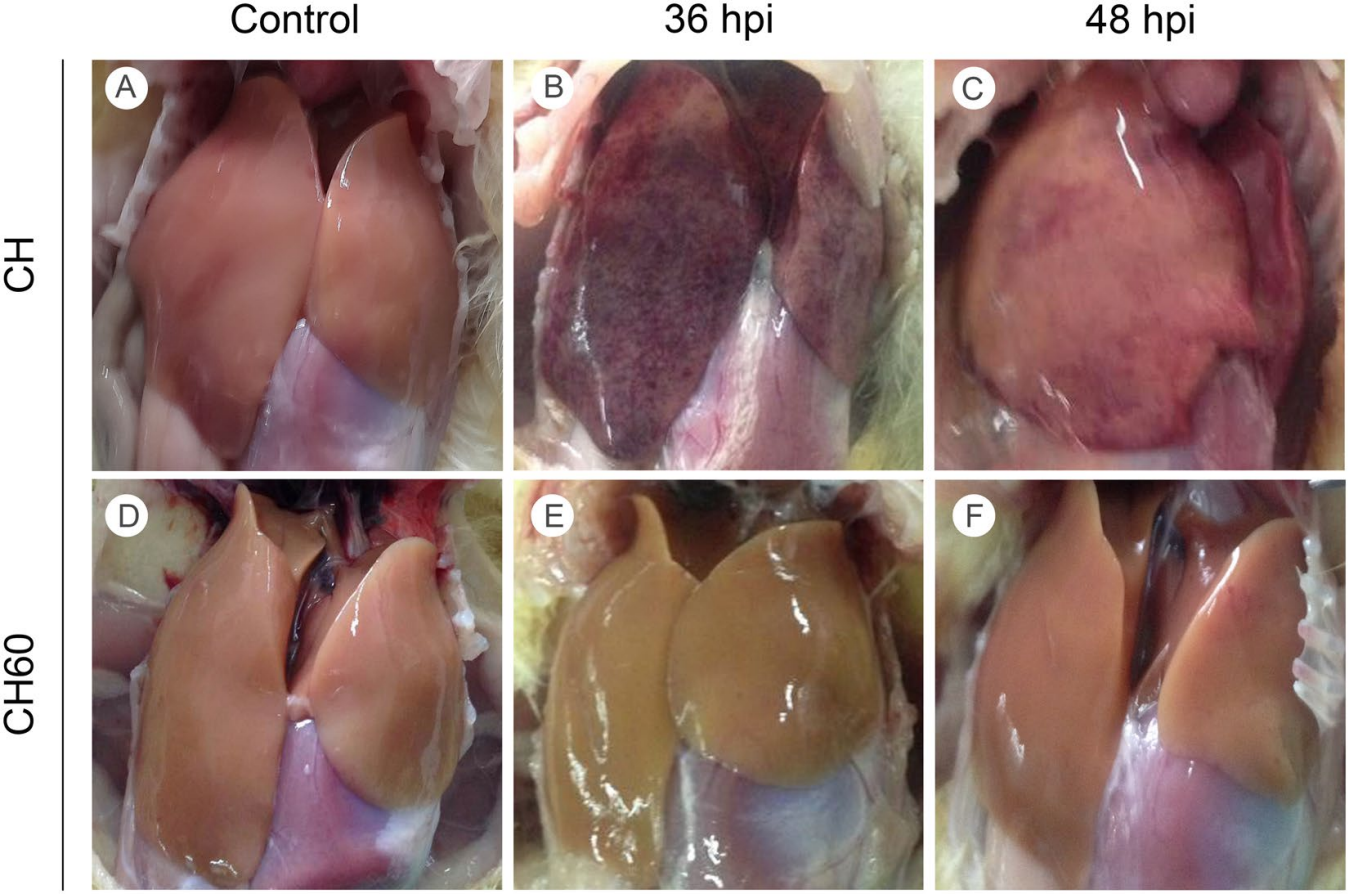
Gross liver lesions in CH and CH60-inoculated ducklings. (**A**,**D**) Livers of the control group, (**B**,**C**) CH-inoculated ducklings at 36 and 48 hpi, (**E**,**F**) and CH60-inoculated ducklings at 36 and 48 hpi.

Post-mortem examination revealed ecchymotic hemorrhage and slight swelling in the livers of CH-infected ducklings at 24 hpi (Supplementary Fig. 1B), while these parameters were enhanced at 36 hpi (Fig. 2B). Lesions in the liver switched from ecchymotic hemorrhages to punctate hemorrhages at 48 hpi (Fig. 2C), and the hemorrhages gradually diminished and eventually disappeared at 60 and 72 hpi (Supplementary Fig. 1C,D). The livers of CH60-inoculated ducklings exhibited no typical gross lesions, and no significant differences in control and CH60-inoculated livers were observed (Fig. 2D–F and Supplementary Fig. 1E–H).

### Histopathological analysis

**Figure 3:**
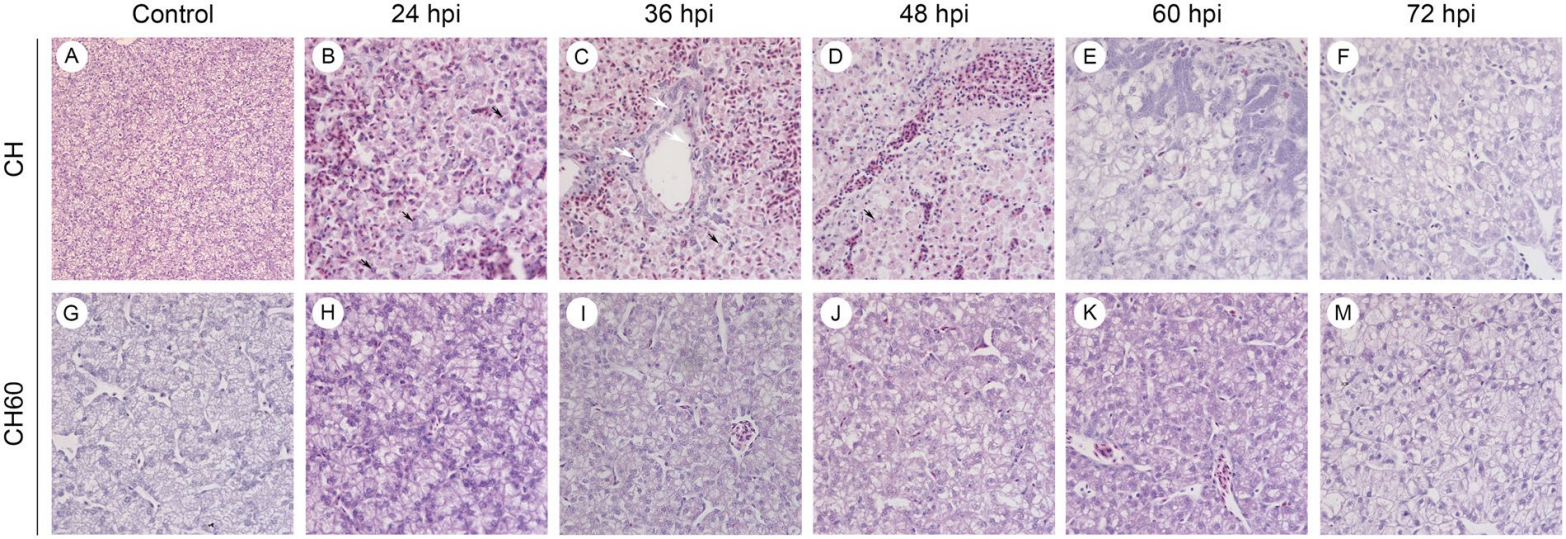
Microscopic lesions in the livers of CH and CH60-inoculated ducklings examined using HE staining. (**A**,**G**) Microscopic lesions in livers of the control group, (**B**–**F**), CH-inoculated ducklings from 24 to 72 hpi, (**H**–**M**) and CH60-inoculated ducklings from 24 to 72 hpi. A, magnification, x200; **B**–**M**, magnification, x600.

Significant differences in the microscopic lesions of CH and CH60-inoculated duckling livers were observed (Fig. 3). In CH-inoculated ducklings, liver parenchymal cells were heavily infiltrated by large numbers of red blood cells, and parts of the cell nuclei underwent pyknosis, karyolysis or karyorhexis. However, small numbers of lymphocytes were found in the hepatic sinusoid at 24 hpi (Fig. 3B). At 36 hpi, hepatocytes were significantly infiltrated by large numbers of red blood cells, accompanied by steatosis, necrosis and hepatic lobule disappearance (Fig. 3C). Additionally, numerous lymphocytes infiltrated the hepatic sinusoid, and cell nuclei underwent pyknosis or karyolysis at 48 hpi (Fig. 3D). Moreover, we observed numerous apoptotic bodies (Fig. 3 black arrows) and a small number of lymphocytes (Fig. 3 white arrows). We also observed the proliferation of bile duct epithelial cells, accompanied by notable steatosis and concentrated cell nuclei (Fig. 3E). No obvious pathological damage was observed in CH60-inoculated ducklings; however, we found scattered lymphocytes distributed in the hepatic sinusoid (Fig. 3H–M).

### Apoptosis in CH and CH60-inoculated duckling livers

**Figure 4:**
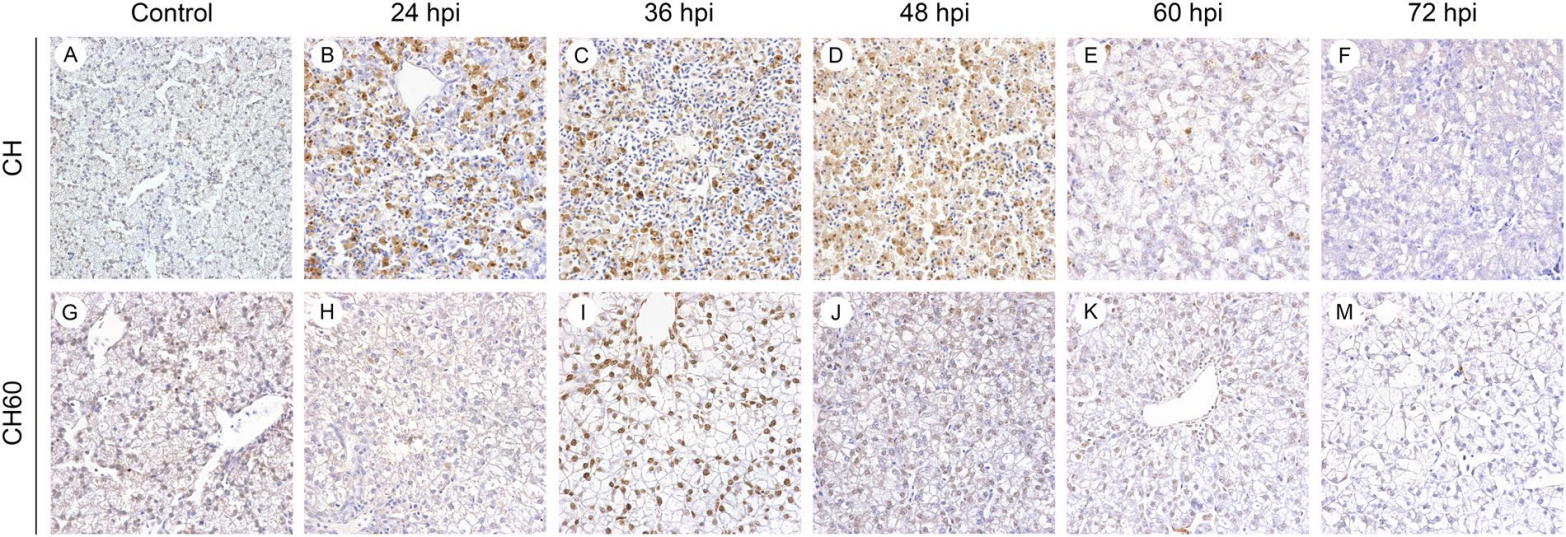
TUNEL assay in the livers of CH and CH60-inoculated ducklings. (**A**,**G**) TUNEL staining in the livers of the control group, (**B**–**F**) CH-inoculated ducklings from 24 to 72 hpi, (**H**–**M**) and CH60-inoculated ducklings from 24 to 72 hpi. A, magnification, x400; **B**–**M**, magnification, x600.

Our group previously confirmed the ability of the GTPase-like 2A2 protein of DHAV-1 to induce apoptosis in primary cell culture, potentially contributing to the DHAV-1 pathogenesis^15^. However, there have been no reports investigating apoptosis in CH- inoculated or CH60-inoculated duckling livers. According to terminal dUTP nick-end labeling (TUNEL) analysis, both the CH and CH60 strains induced apoptosis in the liver. We observed large numbers of apoptotic cells at 24, 36, and 48 hpi in CH-infected duckling livers (Fig. 4B–D), which were associated with microscopic lesions (Fig. 3B–D). Additionally, the CH60 strain induced apoptosis that peaked at 36 hpi (Fig. 4I).

### Viral RNA loads in the livers of CH and CH60-inoculated ducklings

**Figure 5:**
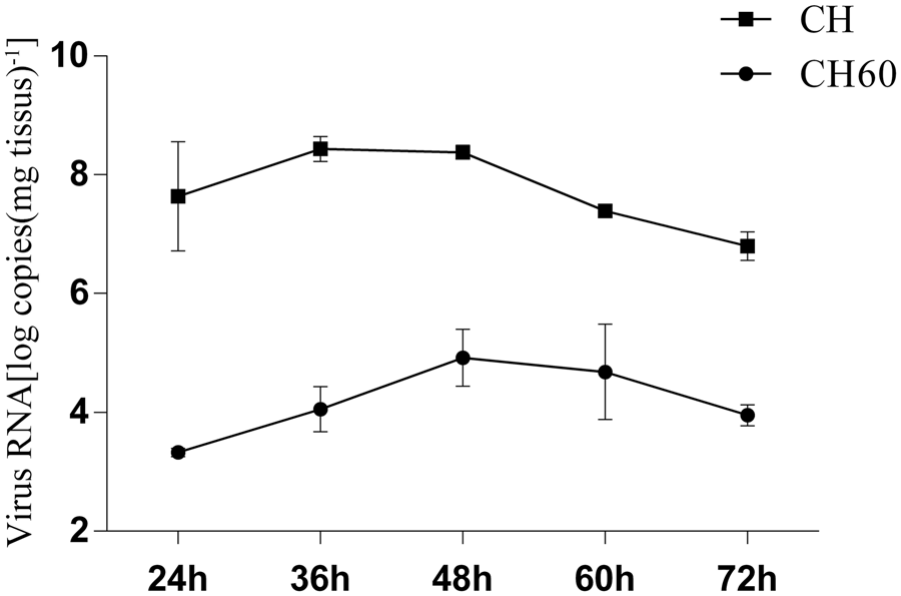
Viral VP0 gene expression. Log of viral RNA copies in the livers of CH and CH60-inoculated ducklings.

To ensure the presence of equivalent numbers of inoculated viruses, viral genomic RNA copies of the CH and CH60 strains were determined by performing quantitative real-time (qRT)-PCR to detect the viral VP0 gene. The number of genomic RNA copies in 1 ml of the CH strain was approximately five-eighths of that in 1 ml of the CH60 strain (data not shown). Thus, ducks in the experimental groups were intramuscularly inoculated with 0.40 ml of the CH strain and 0.25 ml of the CH60 strain. Total RNA was extracted from liver tissue at 24, 36, 48, 60 and 72 hpi, and copy numbers were measured by qRT-PCR. As shown in Fig. 5, viral RNA of the CH strain increased to 2.51 × 10^8^ copies/mg of tissue at 36 hpi, was sustained to 48 hpi, and then began to decrease at 60 hpi. Proliferation of the CH60 strain exhibited a similar phenomenon to that of the CH strain, and CH60 viral RNA peaked at 48 hpi, which was later than that for the CH strain.

### Expression of innate immune-related genes in the livers and blood of DHAV-1-infected ducks

**Figure 6:**
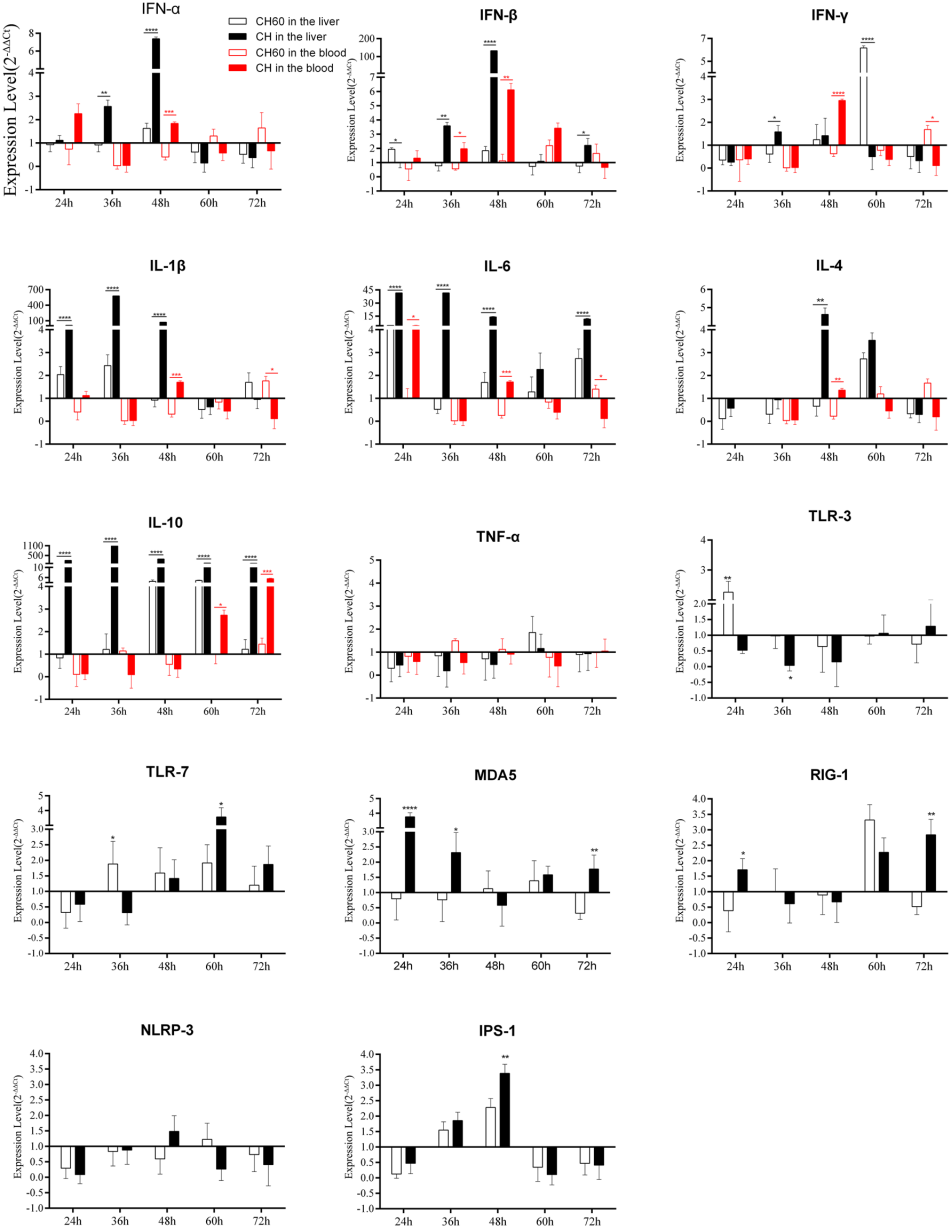
Dynamic changes in immune-related genes in the livers and blood of CH and CH60-inoculated ducklings. The livers and blood of CH and CH60-inoculated ducklings were collected at 24, 36, 48, 60, and 72 hpi. Total RNA was extracted, and cDNA was prepared for cytokine detection. Cytokine expression levels were measured by the 2^−ΔΔCt^ method with relative quantification. Differences in expression levels of the various genes between strains CH and CH60 at each time point (24, 36, 48, 60 and 72 hpi) were analyzed using Student’s *t* test and were considered significant as follows: *P < 0.05; **P < 0.01; ****P < 0.0001.

Innate immunity involves the recognition of invasive pathogenic microorganisms by pattern recognition receptors (PRRs), resulting in the expression of antiviral molecules. PRRs, such as Toll-like receptor 3 (TLR3), TLR7, retinoic acid-inducible gene I (RIG-I) and melanoma differentiation-associated gene 5 (MDA5), recognize viral RNA. Therefore, we measured the expression levels of these four PRRs in the liver. As shown in Fig. 6, the CH strain downregulated TLR3 and TLR7 during the early stage of infection (24 and 36 hpi); this downregulation was most obvious at 36 hpi. During the later stage of infection (60 hpi, 72 hpi), TLR3 and TLR7 expression gradually increased. Similar observations were made regarding the expression of MDA5 and RIG-I. Downregulation of these four PRRs may be related to viral escape mechanisms. The CH strain blocked the expression of host antiviral molecules by inhibiting the expression of PRRs to facilitate its own proliferation, as shown by characterizing viral replication (Fig. 5). Additionally, the CH60 strain up-regulated TLR7 within 36–72 hpi. Subsequently, we detected the expression of IPS-1, a key molecule downstream of MDA5 and RIG-I. IPS-1 was significantly up-regulated at 36–48 hpi with both CH and CH60 and exhibited some hysteresis compared with MDA5 and RIG-I expression, a trend that supported its involvement in host regulatory mechanisms.

IFNs are antiviral molecules that play important roles in clearing invading pathogenic microorganisms^16–18^. We determined the transcriptional levels of IFN-α, IFN-β and IFN-γ, observing up-regulated IFN-α and IFN-β expression at 36 hpi followed by a peak at 48 hpi in the livers of CH-inoculated ducklings (Fig. 6). Similar results were found in blood samples from CH-inoculated ducklings (Fig. 6). Interestingly, IFN-β expression in the livers of CH-inoculated ducklings was 133-fold higher at 48 hpi. Next, pro-inflammatory cytokines (IL-1β, IL-6, and TNF-α) and anti-inflammatory cytokines (IL-4, IL-10) were assessed in the liver and blood samples. The expression levels of IL-1β, IL-6 and IL-10 in the liver were significantly up-regulated after CH infection, peaked at 36 hpi and maintained the same levels until 48 hpi. However, IL-10 expression in the blood was downregulated before 48 hpi. The cytokine storm caused by IFN-α, IFN-β, IL-1β and IL-6 in the livers of CH-inoculated ducklings was related to gross lesions (Fig. 2B–C), microscopic pathological damage (Fig. 3B–D) and apoptosis (Fig. 4B–D). In CH60-inoculated ducklings, the expression of IFN-α, IFN-β, IFN-γ, IL-1β, IL-6 and IL-10 also changed. Remarkably, IFN-γ expression was up-regulated, but IL-10 expression showed no obvious changes, potentially indicating the engagement of adaptive immunity. Changes in IL-4 expression were similar to those observed for TNF-α. Specifically, both the CH and CH60 strains inhibited IL-4 and TNF-α expression during the early stage of infection, and both cytokines were up-regulated after 60 hpi.

## Discussion

Previous studies investigating DHAV-1 have focused on only the viral pathogenicity of a single strain and the resulting host immune responses. In this study, we established an experimental model of infection with the virulent CH DHAV-1 strain and the attenuated CH60 commercial vaccine strain to investigate the pathogenicity of DHAV-1 and immune responses in 7-day-old ducklings. The CH strain caused nearly 50% mortality in ducklings and induced more efficient virus replication in the liver. Massive hemorrhages and necrotic lesions appeared on the liver surface with virus replication of the CH strain, which is consistent with previous reports in the literature^2,19,20^. Additionally, the presence of microscopic lesions revealed liver parenchyma infiltration by large numbers of red blood cells, and this effect was accompanied by hepatocyte necrosis and apoptosis corresponding to hemorrhages and necrotic lesions on the liver surface.

The innate immune system is the first line of defense against invading pathogens, which are recognized by PRRs. Picornaviruses are recognized by three classical PRRs: TLRs, RIG-I-like receptors (RLRs) and nucleotide oligomerization domain (NOD)-like receptors (NLRs)^18^. TLRs are transmembrane proteins that recognize viral components in extracellular and cytoplasmic vacuoles; TLR3 recognizes double-stranded RNA, while TLR7 recognizes single-stranded RNA^21,22^. RLRs constitute a family of cytoplasmic proteins that includes RIG-I, MDA5 and LGP2. RIG-I binds to dsRNA or ssRNA bearing a 5′ triphosphate, and MDA5 recognizes long dsRNA^23,24^. We evaluated the expression of TLR3, TLR7, MDA5, RIG-I and NLRP3 at 24, 36, 48, 60 and 72 hpi. The CH strain significantly inhibited these five PRRs. Therefore, we hypothesized that this phenomenon might be associated with immunological escape mechanisms of the DHAV-1 CH strain. The escape mechanisms of enterovirus^25^, which also belongs to the picornavirus family, have previously been elucidated. For example, the 2 A proteins of enterovirus 71 and coxsackievirus B3 cleave MDA5, and the 3 C protein inhibits the expression of RIG-I^26–28^. According to Barral PM *et al*., poliovirus (PV) depends on caspases and proteasomes to reduce the expression of MDA5 after infecting cells, and PV may block type I IFN production by cleaving MDA5^29^. As shown by Kotla S *et al*.^30^, PV blocks the activation of IRF-3 but does not inhibit MDA-5 or MAVS to inhibit IFN-I expression. However, the escape mechanisms of DHAV-1 have not been elucidated, and subsequent experiments using siRNA knockdown or poly I:C stimulation are required to further confirm the roles of PRRs in DHAV infection and specific escape mechanisms^31^. Expression of the PRRs TLR3, TLR7, RIG-I and NLRP-3 was depressed in the first 48 hours, while that of IL-1β, IL-6 and IL-10 was elevated in the first 48 hpi. Interestingly, IPS-1 (also called MAVS) expression was elevated in the first 48 hours. In the NOD-like receptor signaling pathway, the RNA virus is first recognized by NOD2, and MAVS is then activated, resulting in the elevated expression of IFN-α/β. In addition, the NF-kappa B signaling pathway, which activates IL-1β, IL-6, can be activated via MAVS-dependent and independent mechanisms^32^. Therefore, DHAV-1 may elevate cytokine levels via the NOD-Like receptor signaling pathway.

Apoptosis is a programmed cell death process intended to eliminate cells^33,34^. Hepatocyte apoptosis is an important feature of acute liver injuries and either precedes or exists simultaneously to the onset of necrosis^35^. Therefore, we characterized the apoptosis of CH and CH60-inoculated livers (Fig. 4B–D). The CH strain induced massive hepatocyte apoptosis as well as oncotic necrosis (Fig. 3B–F), which are typical of acute liver injury. TNF-α, a death receptor ligand, initiates apoptosis. However, TNF-α expression was inhibited by both the CH and CH60 strains. Other stimuli that initiate apoptosis include Fas ligand, DNA damage and growth factor withdrawal^36,37]^. X. D. Sheng *et al*.^38^ evaluated apoptosis-related gene expression in the livers of DHAV-1-infected ducklings and observed the significant up-regulation of Bcl-2 transcription, whereas the expression of caspase-3, -8 and -9 was not obviously altered. Therefore, the specific mechanisms underlying DHAV-1-induced apoptosis require further investigation.

A cytokine storm is an excessive immune response stimulated by viruses, bacteria and external factors. A severe cytokine storm produces markedly higher levels of pro-inflammatory cytokines, including IFNs, interleukins, chemokines, and tumor necrosis factors, which are responsible for multi-organ dysfunction, via a specific feedback mechanism^39–41^. Cytokine storms were first discovered in graft-versus-host disease in 1993^42^, and influenza virus, variola virus, and severe acute respiratory syndrome coronavirus (SARS-CoV) were all later found to cause severe cytokine storms^43–45^. However, there have been no reports investigating cytokine storms resulting from DHAV-1 infection. In our study, the IFN-α, IFN-β, IL-1β, and IL-6 transcriptional levels were markedly up-regulated. Furthermore, the liver surface exhibited extensive punctate hemorrhaging, and the liver parenchyma was significantly infiltrated with numerous red blood cells, accompanied by steatosis and necrosis. Similar effects were not observed in CH60-inoculated ducklings, but cytokine storms have been identified in the adult duck kidney^14^ and liver^46^. Therefore, the pathological mechanisms induced by DHAV-1 are likely related to severe cytokine storms. IFN-γ is primarily secreted by Th1 cells and mononuclear macrophages, which are associated with cellular immunity. However, IL-10 is largely secreted by Th2 cells, which inhibit the proliferation and activation of Th1 cells, and monocyte macrophages, thus promoting the proliferation and activation of B lymphocytes and correlating with humoral immunity^47^. Notably, IL-10 was up-regulated after CH strain infection, but IFN-γ expression did not change significantly. The hyper-induction of IL-10 potentially inhibits IFN-γ expression and, in turn, inhibits cellular immunity rather than enhancing humoral immunity. Persistent inflammatory responses cause immune system dysfunction and ultimately cause liver damage.

In conclusion, severe cytokine storms caused by the CH strain induced hemorrhagic liver lesions, resulting in the rapid death of ducklings.

## Materials and Methods

### Ethics statement

This study was approved by the Committee of Experiment Operational Guidelines and Animal Welfare of Sichuan Agricultural University. Experiments were conducted in accordance with approved guidelines.

### Viruses and animals

The DHAV-1 CH strain and the DHAV-1 CH60 attenuated vaccine were provided by the Institute of Preventive Veterinary Medicine at Sichuan Agricultural University. Ducks were infected with the CH strain at a concentration of 10^7.88^ copies/ml and the CH60 strain at a concentration of 10^8.07^ copies/ml as determined by qRT-PCR.

One-day-old Cherry Valley ducks were purchased from the poultry farm of Sichuan Agricultural University and were raised in isolators. The ducks were confirmed to be free of DHAV-1 or IgG against DHAV-1 by one step reverse-transcription PCR^48^ and indirect ELISA^49^ detection in serum samples.

### Experimental procedure

After one week, the ducks were randomly divided into three groups of 15 and raised in separate isolators. The ducks in the first group received 0.40 ml of the DHAV-CH strain (10^7.88^ copies/ml) via intramuscular injection, the ducks in the second group received 0.25 ml of the DHAV-CH60 strain (10^8.07^ copies/ml) via intramuscular injection, and ducks in the last group were injected with 0.25 ml of 0.75% physiological normal saline (NS) as a negative control. Three ducklings from each group were killed at 24, 36, 48, 60 and 72 hpi, and their livers and blood were collected. Fifty-milligram liver specimens were weighed and immediately placed in a solution to protect the RNA and DNA in the samples (code. no 9750, TaKaRa, Japan) until RNA isolation was performed. Additionally, portions of the liver were soaked in 4% paraformaldehyde solution for histopathological examination.

To identify the mortality rates of CH- and CH60-inoculated ducklings, 30 one-week-old ducks were randomly divided into three groups (n = 10) and raised in separate isolators. The ducks in the first group received 0.40 ml of the DHAV-CH strain (10^7.88^ copies/ml) via intramuscular injection, the ducks in the second group received 0.25 ml of the DHAV-CH60 strain (10^8.07^ copies/ml) via intramuscular injection, and the ducks in the last group were injected with 0.25 ml of 0.75% physiological normal saline (NS) as a negative control. Signs of disease and death were observed within one week.

### HE staining and the TUNEL assay

Livers soaked in 4% paraformaldehyde solution were dehydrated, embedded in paraffin, cut into 4-μm-thick sections and stained with hematoxylin and eosin (HE) using standard procedures.

Four-micron sections were also used to perform the TUNEL using an *In-Situ* Apoptosis Detection Kit (Boster Inc., Wuhan, China) according to the manufacturer’s instructions. Apoptotic cells were observed under a light microscope.

### RNA isolation and cDNA preparation

Total RNA was isolated from 50 mg of liver and 200 µL of blood specimens using RNAiso Plus Reagent (TaKaRa) according to the manufacturer’s instructions. Genomic DNA was then removed, and reverse transcription was performed using a PrimeScript^™^ RT Reagent Kit (Perfect Real Time, TaKaRa) according to the manufacturer’s instructions.

### Viral RNA load in the liver and cytokine expression in the liver and blood

Viral copies in total RNA were measured using methods previously established in our laboratory^50,51^. Fourteen immune-related genes (TLR3, TLR7, NLRP3, MDA5, RIG-I, IPS-1, IL-1β, IL-4, IL-6, IL-10, TNF-α, IFN-α, IFN-β, IFN-γ) and a housekeeping gene (β-actin) were detected by qPCR using previously published primer sequences as well as newly designed primer sequences for TLR3, NLRP3, MDA5, RIG-I, IPS-1, IL-4, IL-10, TNF-α, and IFN-β using Primer Premier 5 (Table 1). The expression levels of immune-related genes were determined by qPCR using a SYBR®Premix Ex Taq™ II (Tli RNaseH Plus) Kit (Takara) and an Applied CFX96 Real-Time PCR Detection System (Bio-Rad, Hercules, CA, USA). Amplification was performed in 10-µl reaction volumes containing 0.5 µl of each primer and 1 µl of RNA. The following thermal cycling conditions were applied: initial activation at 95 °C for 30 s, 40 cycles of denaturation at 95 °C for 5 s and annealing and extension at 58.6 °C for 30 s, and a dissociation curve analysis step.

**Table 1:** Primer sequences used for gene expression profiling.

Gene	Forward (5′–3′)	Reverse (5′–3′)	AD	Reference
IFN-α	TCCACCTCCTCCAACACCTC	TGGGAAGCAGCGCTCGAG	AY879230.1	^52^
IFN-β	CCTCAACCAGATCCAGCATT	GGATGAGGCTGTGAGAGGAG	AY831397	^52^
IFN-γ	GCTGATGGCAATCCTGTTTT	GGATTTTCAAGCCAGTCAGC	AJ012254	^52^
TLR3	AACACTCCGCCTAAGTATCAT	CTATCCTCCACCCTTCAAAA	JN573268	new
TLR7	CCTTTCCCAGAGAGCATTCA	TCAAGAAATATCAAGATAATCACATCA	AY940195	^52^
MDA5	CTGCCCGCTACTTGAACTCCA	GCACCATCTCTGTTCCCACGA	KJ451070.1	new
RIG-I	GCGTACCGCTATAACCCACA	CCTTGCTGGTTTTGAACGC	AB772012.1	new
NLRP-3	CATCCCAGTGAAGCGTTGA	GCCATCTGGTCGTATAGCG	K12800	new
IL-1β	TCGACATCAACCAGAAGTGC	GAGCTTGTAGCCCTTGATGC	DQ393268	^52^
IL-4	TACCTCAACTTGCTGCACATC	GCTACTCGTTGGAGGGTTCT	K05430	new
IL-6	TTCGACGAGGAGAAATGCTT	CCTTATCGTCGTTGCCAGAT	AB191038	^52^
IL-10	AGCAGCGAGCACCACCA	TGCCGTTCTCGTTCATCTTT	K05443	new
TNF-α	TTTTATGACCGCCCAGTT	TAGGCAGAGGCCACCA	K19363	new
IPS-1	CTTCGGGAACTCCAAACACCT	TGCCTCCCCTGAGATCCTG	KJ466052.1	new
β-actin	TACGCCAACACGGTGCTG	GATTCATCATACTCCTGCTTG	EF667345.1	^52^

### Statistical analysis

All statistical and imaging analyses were performed using GraphPad Prism 6 software. The relative mRNA expression of target genes was analyzed using the 2^−ΔΔCt^ method and compared with that in the control group injected with 0.25 ml of NS. ΔCt values were determined by subtracting the average Ct values of the endogenous control gene β-actin from those of the target genes.

## Electronic supplementary material

Supplementary Information 
