# Author Correction: Cytokine storms are primarily responsible for the rapid death of ducklings infected with duck hepatitis A virus type 1

**DOI:** 10.1038/s41598-020-62439-4

**Published:** 2020-03-24

**Authors:** Jinyan Xie, Mingshu Wang, Anchun Cheng, Xin-Xin Zhao, Mafeng Liu, Dekang Zhu, Shun Chen, Renyong Jia, Qiao Yang, Ying Wu, Shaqiu Zhang, Yunya Liu, Yanling Yu, Ling Zhang, Kunfeng Sun, Xiaoyue Chen

**Affiliations:** 1 Institute of Preventive Veterinary Medicine, Sichuan Agricultural University, Wenjiang, Chengdu City, Sichuan People’s Republic of China; 2 Key Laboratory of Animal Disease and Human Health of Sichuan Province, Sichuan Agricultural University, Wenjiang, Chengdu City, Sichuan People’s Republic of China; 3 Avian Disease Research Center, College of Veterinary Medicine, Sichuan Agricultural University, Wenjiang, Chengdu City, Sichuan People’s Republic of China

Correction to: *Scientific Reports* 10.1038/s41598-018-24729-w, published online 26 April 2018

This Article contains errors in the Materials and Methods section, under the subheading ‘Viral RNA load in the liver and cytokine expression in the liver and blood’.

“Amplification was performed in 10-µl reaction volumes containing 0.5 µl of each primer and 1 µl of RNA”

should read:

“Amplification was performed in 10-µl reaction volumes containing 0.5 µl of each primer and 1 µl of cDNA”

In addition, this Article contains errors in Reference 46 which was incorrectly given as:

Ou, X. *et al*. The neglected avian hepatotropic virus induces acute and chronic hepatitis in ducks: an alternative model for hepatology. *Oncotarget*, (2017).

The correct reference is listed below as ref. 1:
