# Supplementary material for: Cytokine storms are primarily responsible for the rapid death of ducklings infected with duck hepatitis A virus type 1
Source: Sci Rep. 2018 Apr 26;8:6596. doi: 10.1038/s41598-018-24729-w (PMC5920089; doi:10.1038/s41598-018-24729-w)
Supplement: Supplementary file 1 — Supplementary Information [file 41598_2018_24729_MOESM1_ESM.docx]

**Cytokine storms are primarily responsible for the rapid death of ducklings infected with duck hepatitis A virus type 1**

Jinyan Xie^1,2¶^, Mingshu Wang^1,2,3¶^, Anchun Cheng^1,2,3*^, Xin-Xin Zhao^1,2,3^, Mafeng Liu^1,2,3^, Dekang Zhu^2,3^, Shun Chen^1,2,3^, Renyong Jia^1,2,3^, Qiao Yang^1,2,3^, Ying Wu^1,2,3^, Shaqiu Zhang^1,2,3^, Yunya Liu^1,2,3^, Yanling Yu^1,2,3^, Ling Zhang^1,2,3^, Kunfeng Sun^1,2,3^, Xiaoyue Chen^2,3^

^1^Institute of Preventive Veterinary Medicine, Sichuan Agricultural University, Wenjiang, Chengdu City, Sichuan, People’s Republic of China

^2^Key Laboratory of Animal Disease and Human Health of Sichuan Province, Sichuan Agricultural University, Wenjiang, Chengdu City, Sichuan, People’s Republic of China

^3^Avian Disease Research Center, College of Veterinary Medicine, Sichuan Agricultural University, Wenjiang, Chengdu City, Sichuan, People’s Republic of China

^¶^These authors contributed equally to this work as first authors.

*Corresponding authors.

E-mail: [chenganchun@vip.163.com](mailto:chenganchun@vip.163.com) (Anchun Cheng)


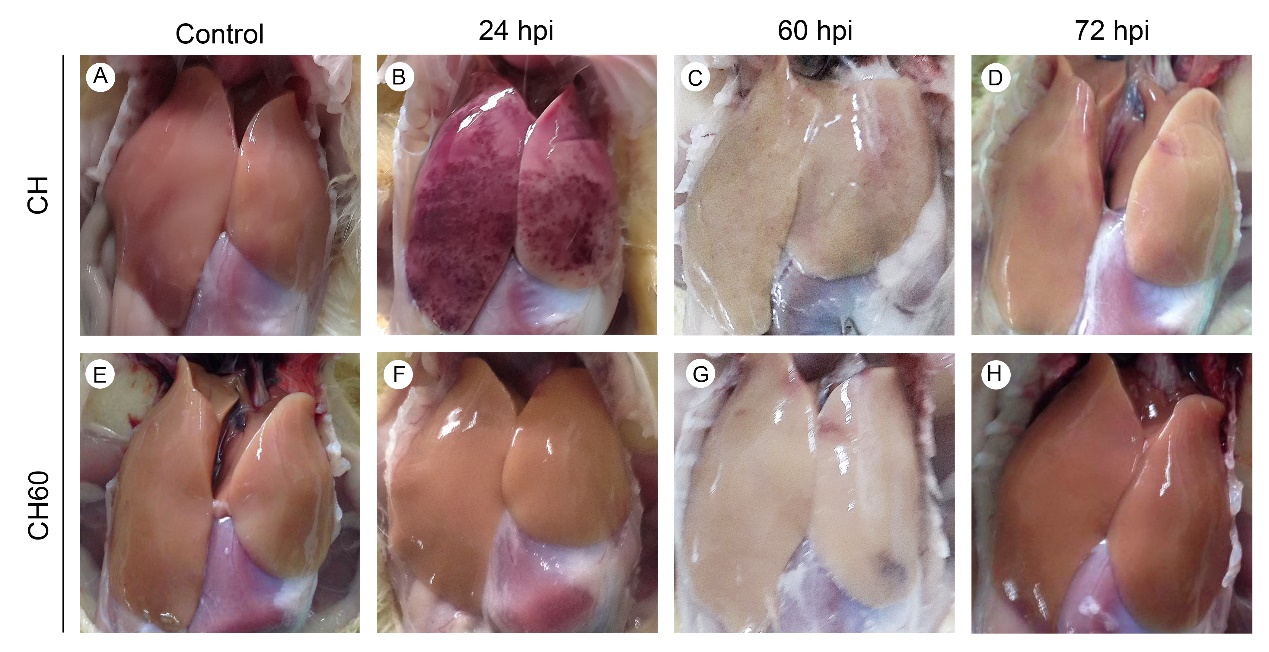


Supplementary Figure 1. Gross liver lesions in CH-infected or CH60-immunized ducklings. (A, E) Livers of the control group, (B-D) livers of CH-infected ducklings at 24, 60, and 72 hpi, and (F-H) livers of CH60-immunized ducklings at 24, 60, and 72 hpi.


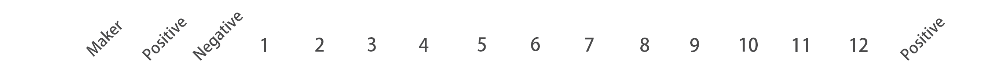
A


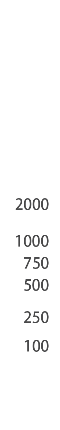

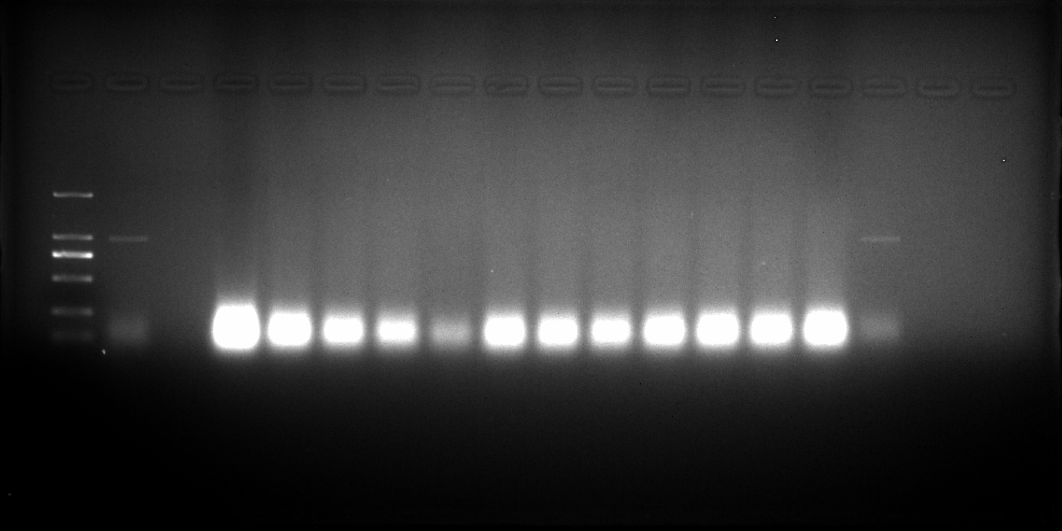


B





Supplementary Figure 2. (A) The detection of serological status for DHAV-1. Line 1-12 represented 12 serum samples, respectively. Positive samples for DHAV-1 was 992 bp. (B) The detection of IgG against DHAV-1. DHAV-1 positive and negative serum, and 8 serum samples were adopted to test IgG, using indirect ELISAs. The dashed line represents the cutoff value.
